# Supplementary material for: Pyruvate Dehydrogenase Kinase 1 inhibition mediated oxidative phosphorylation enhancement in cartilage promotes osteoarthritis progression
Source: BMC Musculoskelet Disord. 2023 Jul 20;24:597. doi: 10.1186/s12891-023-06585-6 (PMC10357736; doi:10.1186/s12891-023-06585-6)
Supplement: Supplementary file 1 — Additional file 1. [file 12891_2023_6585_MOESM1_ESM.zip › MF.Gsea.1653627316242/GOMF_BICARBONATE_TRANSMEMBRANE_TRANSPORTER_ACTIVITY.html]

Details for gene set GOMF\_BICARBONATE\_TRANSMEMBRANE\_TRANSPORTER\_ACTIVITY[GSEA]

|  || Dataset | 55457\_collapsed\_to\_symbols.cls.cls #OA\_versus\_normal.cls.cls #OA\_versus\_normal\_repos |
| Phenotype | cls.cls#OA\_versus\_normal\_repos |
| Upregulated in class | normal |
| GeneSet | GOMF\_BICARBONATE\_TRANSMEMBRANE\_TRANSPORTER\_ACTIVITY |
| Enrichment Score (ES) | -0.5792428 |
| Normalized Enrichment Score (NES) | -1.6477618 |
| Nominal p-value | 0.022108844 |
| FDR q-value | 0.13068865 |
| FWER p-Value | 0.975 |
Table: GSEA Results Summary

  

Fig 1: Enrichment plot: GOMF\_BICARBONATE\_TRANSMEMBRANE\_TRANSPORTER\_ACTIVITY      
 Profile of the Running ES Score & Positions of GeneSet Members on the Rank Ordered List

  

| SYMBOL | TITLE | RANK IN GENE LIST | RANK METRIC SCORE | RUNNING ES | CORE ENRICHMENT || 1 | SLC4A1 | solute carrier family 4 member 1 (Diego blood group) [Source:HGNC Symbol;Acc:HGNC:11027] | 3233 | 0.227 | -0.2031 | No |
| 2 | SLC26A4 | solute carrier family 26 member 4 [Source:HGNC Symbol;Acc:HGNC:8818] | 4552 | 0.109 | -0.2824 | No |
| 3 | SLC4A4 | solute carrier family 4 member 4 [Source:HGNC Symbol;Acc:HGNC:11030] | 5027 | 0.071 | -0.3043 | No |
| 4 | SLC39A8 | solute carrier family 39 member 8 [Source:HGNC Symbol;Acc:HGNC:20862] | 6450 | -0.039 | -0.4066 | No |
| 5 | SLC4A8 | solute carrier family 4 member 8 [Source:HGNC Symbol;Acc:HGNC:11034] | 6455 | -0.039 | -0.3987 | No |
| 6 | SLC4A5 | solute carrier family 4 member 5 [Source:HGNC Symbol;Acc:HGNC:18168] | 6572 | -0.047 | -0.3978 | No |
| 7 | SLC26A1 | solute carrier family 26 member 1 [Source:HGNC Symbol;Acc:HGNC:10993] | 7618 | -0.128 | -0.4518 | No |
| 8 | SLC26A3 | solute carrier family 26 member 3 [Source:HGNC Symbol;Acc:HGNC:3018] | 9261 | -0.253 | -0.5261 | Yes |
| 9 | SLC26A2 | solute carrier family 26 member 2 [Source:HGNC Symbol;Acc:HGNC:10994] | 9523 | -0.276 | -0.4882 | Yes |
| 10 | SLC26A6 | solute carrier family 26 member 6 [Source:HGNC Symbol;Acc:HGNC:14472] | 9898 | -0.311 | -0.4518 | Yes |
| 11 | BEST1 | bestrophin 1 [Source:HGNC Symbol;Acc:HGNC:12703] | 10469 | -0.370 | -0.4182 | Yes |
| 12 | CFTR | CF transmembrane conductance regulator [Source:HGNC Symbol;Acc:HGNC:1884] | 10920 | -0.420 | -0.3646 | Yes |
| 13 | SLC4A3 | solute carrier family 4 member 3 [Source:HGNC Symbol;Acc:HGNC:11029] | 11318 | -0.474 | -0.2957 | Yes |
| 14 | SLC4A7 | solute carrier family 4 member 7 [Source:HGNC Symbol;Acc:HGNC:11033] | 11587 | -0.513 | -0.2085 | Yes |
| 15 | SLC26A10 | solute carrier family 26 member 10 [Source:HGNC Symbol;Acc:HGNC:14470] | 12899 | -1.475 | 0.0001 | Yes |
Table: GSEA details [plain text format]

  

Fig 2: GOMF\_BICARBONATE\_TRANSMEMBRANE\_TRANSPORTER\_ACTIVITY      
 Blue-Pink O' Gram in the Space of the Analyzed GeneSet

  

Fig 3: GOMF\_BICARBONATE\_TRANSMEMBRANE\_TRANSPORTER\_ACTIVITY: Random ES distribution      
 Gene set null distribution of ES for **GOMF\_BICARBONATE\_TRANSMEMBRANE\_TRANSPORTER\_ACTIVITY**
